# Comparing the new concept of impairment in personality functioning with borderline personality disorder: differential psychosocial and psychopathological correlates in a clinical adolescent sample

**DOI:** 10.1007/s00787-024-02555-y

**Published:** 2024-08-16

**Authors:** Andrea Wyssen, Stefan Lerch, Corinna Reichl, Ines Mürner-Lavanchy, Christine Sigrist, Selina Schär, Marialuisa Cavelti, Julian Koenig, Michael Kaess

**Affiliations:** 1 University Hospital of Child and Adolescent Psychiatry and Psychotherapy, University of Bern, Bern, Switzerland; 2 Department of Child and Adolescent Psychiatry, Psychosomatics and Psychotherapy, Faculty of Medicine, University Hospital Cologne, University of Cologne, Cologne, Germany; 3 Department of Child and Adolescent Psychiatry, Center for Psychosocial Medicine, University Hospital Heidelberg, Heidelberg, Germany

**Keywords:** Level of Personality Functioning Scale (LPFS), Alternative model for personality disorders in DSM-5 (AMPD), Borderline personality disorder (BPD), Adolescence

## Abstract

Borderline personality disorder (BPD) is an established diagnosis in adolescence with high comorbidity and psychosocial impairment. With the introduction of the alternative model for personality disorders in DSM-5 (AMPD), personality functioning is operationalized using the Level of Personality Functioning Scale (LPFS), which has been shown to be associated with severity of personality pathology. The present study aimed at examining differential psychopathological and psychosocial correlates of LPFS and BPD. A total of 526 adolescent in- and outpatients were interviewed with the STiP-5.1 (LPFS) and the SCID-II. Mixed linear regression was used to investigate the associations between the two interviews with measures of psychopathology and psychosocial impairment. 11.4% met the diagnostic threshold of both interviews, 16.1% only of the LPFS, and 64.1% were below the diagnostic threshold in both interviews (no PD). The BPD only group was larger than expected—8.4% of patients who met criteria for BPD did not fulfill criteria for significant impairment in the LPFS. The highest burden was found in individuals concurrently showing significant impairment in LPFS and fulfilling BPD diagnosis (LPFS + BPD). Differences between the LPFS only group and the BPD only group were found in risk behavior and traumatic experiences, with higher prevalence in the BPD group. Findings confirm the high psychopathological burden and psychosocial impairment associated with both BPD and LPFS. Those exceeding the diagnostic threshold of LPFS in combination with a BPD diagnosis are characterized by greatest disability. Not all adolescents fulfilling formal BPD diagnosis showed a clinically significant impairment in LPFS, which may refer to a distinct diagnostic group.

**Supplementary Information:**

The online version contains supplementary material available at 10.1007/s00787-024-02555-y.

## Introduction

Borderline personality disorder (BPD) is a severe mental disorder. Comparing treatment seeking adolescents with and without BPD diagnosis indicates significantly higher rates of internalizing and externalizing comorbid mental disorders in the BPD group [1–3]. There is evidence of serious acute symptoms such as high rates of risk-taking behavior, non-suicidal self-injury (NSSI), substance abuse, and suicidality as early symptoms of BPD in adolescence [4–6]. Difficulties in emotion regulation and experiential avoidance [7], as well as high levels of experienced stress have also been associated with BPD in adolescents [8]. Further, adverse childhood experiences (emotional and physical neglect and/or abuse) have been reported (more) commonly in young individuals with BPD [9, 10]. Additionally, in adolescents with BPD, quality of life is rated lower, and impairments in psychosocial functioning are higher, than in adolescents suffering from other mental disorders [5, 11].

A solid empirical evidence base supports the clinical practice of early diagnosis and intervention in adolescents with BPD [e.g., 12, 13]. Consequently, international guidelines for diagnosis and treatment of BPD recommend the assessment and diagnosis of BPD from the age of 12 years [14–16]. However, despite these guidelines, health care professionals are still reluctant to (and therefore, rarely) diagnose personality disorders (PDs) in adolescents; with skepticism and caution prevailing, mostly due to fear of stigma for their patients alongside with continuous doubts regarding the stability and validity of PDs in adolescence [17–21]. This is in contrast to studies that have shown strong evidence of the usefulness and importance of assessment of subthreshold and full-syndrome BPD, by demonstrating that subthreshold forms are similarly associated with psychopathology and impairment [e.g., 5, 22]. BPD has been shown to have the highest reliability and validity among all PD diagnoses [13, 23], which has been recognized in the most recent revisions of the ICD-11 [24] and DSM-5 [25]. That is, BPD is the only PD which is still specifiable in ICD-11. The disorder ranks at the high end of the severity dimension of PD and is closest to a general PD factor [26].

Based on substantiated points of criticism regarding the categorical classification of PD, such as lack of evidence for ten distinct diagnoses, arbitrary diagnostic thresholds, a high degree of overlap between different diagnostic categories, large heterogeneity within categories, and a large proportion of individuals who do not meet the criteria of a specific PD but of an “other specified” or “unspecified” PD [27–30], the conceptualization and classification of PD is undergoing a transition. The new classification of PD in the Alternative Model for Personality Disorders (AMPD) in Section III of the DSM-5 [25] focuses on the global level of severity of impairment in self- and interpersonal functioning as criterion A—the Level of Personality Functioning Scale (LPFS)—and provides five pathological personality traits as criterion B. Similar to the AMPD, ICD-11 operationalizes severity of a PD in terms of impairment in self- and interpersonal functioning. It further provides a list of emotional, cognitive, and behavioural manifestations of personality dysfunction that helps to determine PD severity and expands the AMPD of personality dysfunction [31]. Notably, self-harming behaviour, which is a prominent feature of BPD in adolescents, is described as an aspect of behavioural manifestations. Additionally, the ICD-11 classification contains five trait qualifiers and a borderline pattern qualifier with nine features identical to the DSM-5 BPD criteria [24, 31, 32]. Unlike the former categorical approach with a defined threshold for diagnosis, AMPD and ICD-11 base their classification on a global evaluation of severity and impairment. The alternative model allows a dimensional description of personality functioning and personality traits in individuals with or without PD diagnosis [33]. The dimensional classification has previously been shown to be predictive of course and outcome of PD [34, 35], and is suggested as advantageous regarding the assessment of subtle maladaptive indicators of personality pathology at younger age [36]. The LPFS has shown to predict the existence of a PD according to the categorical diagnostic system, and clinicians have been able to accurately and reliably identify PD pathology if the impairment in personality functioning was at least on a moderate level [35]. Beyond that, the level of impairment in the four elements identity, self-direction, empathy, and intimacy, provides the clinician with relevant additional information and specific patterns or subtypes may refer to meaningful qualitative differences and associated clinical implications [37]. The LPFS is also found to be a significant predictor of comorbidity and psychosocial functioning [38, 39]. Nevertheless, continuity of the categorical approach in the current phase of transition is important, since convergent validity of the categorical system and the AMPD is high for BPD, but relatively low for some other specific PDs (e.g. narcissistic PD) [40].

A recent study provides first evidence for the reliability and validity of the LPFS in adolescents. In a sample of 96 adolescents (n = 84 clinical, n = 12 community) aged 12–17 years, more pronounced self-reported personality problems and symptom severity of general psychopathology were associated with more severe impairment in personality functioning. Moreover, the number of fulfilled BPD criteria was significantly positively correlated with scores on the two domains self- and interpersonal functioning (*r* = 0.29 respectively *r* = − 0.38) [41].

In sum, both BPD and impairments in personality functioning are associated with severe psychopathology in adolescents. However, the LPFS has rarely been investigated in adolescents, and little is known about possible differences in groups of adolescents that are characterized by fulfilling either the clinical threshold of the LPFS or of BPD (or both). Due to the current transition phase of the diagnosis and classification of PDs, it is important to investigate and compare both the categorical and the dimensional system in one study. Thus, the aim of the present study was twofold: *First,* we investigated the concordance between impairment in personality functioning (LPFS) and BPD, expecting a partial overlap. Specifically, that most, if not all adolescents who fulfill the diagnostic threshold for BPD would also exceed the diagnostic threshold of the LPFS, but not vice versa. *Second,* we examined psychopathological and psychosocial correlates of BPD, LPFS and the combination of both, expecting (a) significantly more severe psychopathology and psychosocial impairments in adolescents with BPD and with clinically significant impairment in personality functioning (LPFS) compared to clinical controls without personality pathology, and (b) the highest level of psychopathology and psychosocial impairment in adolescents who exceed the diagnostic threshold of LPFS in combination with a specific BPD diagnosis.

## Methods

### Sample and procedure

For the current analyses, the data of two cohort studies conducted between November 2018 and March 2022 at the University Hospital of Child and Adolescent Psychiatry and Psychotherapy Bern in Switzerland were merged (N = 526). Sample 1 (Bernese Basic Documentation, BeBaDoc) includes consecutive data from adolescent inpatient/day-care treatment (n = 280), and sample 2 (specialized service for adolescents at risk for BPD, AtR!Sk) includes consecutive data from outpatient treatment (n = 246). Inclusion criteria were: 11–18 years of age (BeBaDoc sample), 12–17 years of age (AtR!Sk sample), and sufficient fluency in German language skills. Exclusion criteria were: patients lacking capacity to understand study details or provide informed consent. Data assessment took place within the initial diagnostic phase of outpatient or inpatient treatment. Sample characteristics are presented in Table 1.

**Table 1:** Participant characteristics and differences between samples

	Total sample (N = 526) M (SD)	Sample 1 (inpatient, n = 280) M (SD)	Sample 2 (outpatient, n = 246) M (SD)	Differences (z, χ^2^, p)
*Demographic information*				
Gender (female, %)	405 (78.9)	197 (73.8)	208 (84.6)	χ^2^ = 8.94, *p* = 0.003
Age in years	15.41 (1.52)	15.31 (1.43)	15.51 (1.62)	z = 1.45, *p* = 0.148
Body Mass Index	21.63 (4.92)	21.99 (5.85)	21.26 (3.67)	z = − 0.20, *p* = 0.841
Age at first contact	12.09 (3.38)	12.11 (3.17)	12.07 (3.58)	z = 0.16, *p* = 0.874
*Psychopathology and related factors*				
LPFS facets ≥ 2	4.14 (3.27)	4.70 (3.53)	3.55 (2.87)	z = − 4.70, *p* < 0.001
LPFS threshold (%)	138 (27.5)	86 (33.5)	52 (21.2)	χ^2^ = 9.43, *p* = 0.002
LPFS identity	1.68 (0.95)	1.80 (1.05)	1.54 (0.82)	z = − 3.13, *p* = 0.002
LPFS self-direction	1.30 (0.96)	1.28 (0.97)	1.21 (0.95)	z = − 2.05, *p* = 0.040
LPFS empathy	0.85 (0.78)	0.92 (0.85)	0.76 (0.69)	z = − 1.58, *p* = .113
LPFS intimacy	0.87 (0.87)	0.98 (0.98)	0.74 (0.71)	z = − 1.95, *p* = 0.051
LPFS self-functioning	1.49 (0.86)	1.59 (0.92)	1.38 (0.78)	z = − 2.77, *p* = 0.006
LPFS interpersonal functioning	0.86 (0.74)	0.95 (0.82)	0.75 (0.63)	z = − 2.15, *p* = 0.031
LPFS total	1.17 (0.72)	1.27 (0.79)	1.06 (0.62)	z = − 2.76, *p* = 0.006
BPD criteria	2.69 (2.29)	2.87 (2.42)	2.51 (2.12)	z = − 1.35, *p* = .176
BPD threshold (%)	99 (19.5)	58 (22.2)	41 (16.7)	χ^2^ = 2.49, *p* = .115
Diagnoses MINI-KID	2.74 (2.28)	3.04 (2.43)	2.39 (2.03)	z = − 2.88, *p* = 0.004
SOFAS/ CGAS	56.47 (14.90)	50.37 (14.56)	63.05 (12.25)	z = 9.90, *p* < 0.001
KIDSCREEN-10	19.17 (6.68)	20.20 (7.43)	18.06 (5.55)	z = − 3.01, *p* = 0.003
NSSI year (SITBI-G)	56.78 (91.30)	54.75 (100.52)	58.93 (80.56)	z = 6.13, *p* < 0.001
Suicidal ideation week (SITBI-G)	2.56 (3.74)	2.58 (2.95)	2.54 (4.44)	z = − 0.39, *p* = .697
Suicide attempts year (SITBI-G)	1.54 (8.44)	1.98 (11.39)	1.07 (2.99)	z = − 0.02, *p* = .983
Risk behavior	0.93 (1.14)	0.83 (1.09)	1.03 (1.18)	z = 1.83, *p* = 0.067
CDRS-R	51.82 (16.32)	54.14 (17.80)	49.35 (14.21)	z = − 3.35, *p* = 0.001
PSS-10	26.03 (6.61)	24.77 (7.51)	27.37 (5.16)	z = 3.49, *p* = 0.001
DERS-16	59.48 (13.31)	61.53 (14.01)	57.27 (12.17)	z = − 4.31, *p* < 0.001
CTQ	1.99 (0.53)	2.24 (0.34)	1.72 (0.79)	z = − 11.42, *p* < 0.001

*BPD* Borderline Personality Disorder, *LPFS* Level of Personality Functioning Scale, *MINI-KID* Mini-International Neuropsychiatric Interview for Children and Adolescents, *SOFAS* Social and Occupational Functioning Assessment Scale, *CGAS* Children's Global Assessment Scale, *KIDSCREEN-10* Health Related Quality of Life, *SITBI-G* Self-Injurious Thoughts and Behavior Interview–German Version, *CDRS-R* Children’s Depression Rating Scale—Revised, *PSS-10* Perceived Stress Scale, *DERS-16* Difficulties in Emotion Regulation Scale, 16-item version, *CTQ* Childhood Trauma Questionnaire, *NSSI* Non-Suicidal Self-Injury

Participation rate (informed consent) was 87.8% in sample 1 and 89.0% in sample 2. Specially trained interviewers (post-graduate psychologists) conducted semi-structured interviews. Self-report questionnaires were provided online. The study protocols were approved by the cantonal Ethics Committee (sample 1 ethics ID: 2018-01339, sample 2 ethics ID: 2018-00942) and conforms to the Declaration of Helsinki. All participants were informed in accordance with the study protocol. Written informed consent was obtained from all participants, as well as from a parent or legal guardian for those under the age of 14 years.

### Instruments

#### Interviews

##### Semi-structured interview for personality functioning DSM-5 [STiP-5.1; 42]

The STiP-5.1 is a semi-structured interview designed to assess Criterion A (LPFS) of the AMPD. It assesses the level of impairment in self- and interpersonal functioning with two elements each (self-functioning: identity and self-direction; interpersonal functioning: empathy and intimacy). Each element contains three facets that are rated on a scale ranging from 0–4 (0 = healthy/adaptive functioning, 1 = some impairment, 2 = moderate impairment, 3 = severe impairment, 4 = extreme impairment). According to the AMPD, the diagnostic threshold for a specific PD or a PD trait specified is met if two or more of the four elements have a value of two or higher (i.e., the three facets have a mean of ≥ 2). The STiP-5.1 interview has shown good interrater and re-test reliability, as well as construct validity in clinical and non-clinical samples of adults [43, 44]. Feasibility, reliability and validity of the STiP-5.1 have also been demonstrated in an adolescent sample [41]. Internal consistency (Cronbach’s *α*) of the STiP-5.1 total score in the present sample was *α* = 0.87 and ranged from *α* = 0.68 to *α* = 0.76 for the four elements.

##### Structured clinical interview for DSM-IV Axis II—German version [SCID-II; 45]

The SCID-II is a structured clinical interview to assess PD according to DSM-IV criteria. In the present study, the BPD section was used (9 items; if ≥ 5 criteria were fulfilled for the period of at least one year, BPD was diagnosed) [46].

##### Mini-International neuropsychiatric interview for children and adolescents [MINI-KID; 47]

The number and type of current mental disorders according to DSM-IV and ICD-10 was assessed via the structured interview MINI-KID.

##### Children’s depression rating scale—revised [CDRS-R; 47]

The CDRS-R is a semi-structured interview assessing the severity of depression in childhood and adolescence. Internal consistency (Cronbach’s *α*) in the present sample was 0.89.

##### Global level of functioning (SOFAS and CGAS)

Both scales, the Social and Occupational Functioning Assessment Scale [SOFAS; 48] and the Children's Global Assessment Scale [CGAS; 49] assess the patient’s overall level of functioning in social and occupational areas, independent of the severity of psychopathology.

##### Risk behavior

The variable risk behavior was composed of four areas: (1) alcohol abuse or addiction, (2) substance abuse or addiction according to the MINI-KID, (3) pathological internet use (items assessing the DSM-5 criteria), and (4) regular smoking, with a maximum value of 4.

##### Self-Injurious thoughts and behavior interview—German version [SITBI-G; 50]

The SITBI-G is a semi-structured interview used to assess NSSI, suicidal ideation and suicide attempts.

#### Questionnaires

##### Difficulties in emotion regulation scale, 16-item version [51]

The DERS-16 assesses difficulties in emotion regulation. Internal consistency (Cronbach’s *α*) in the present sample was 0.94.

##### Perceived stress scale [PSS-10; 52]

The PSS-10 contains 10 items to assess the degree to which individuals perceive situations in their life as overloaded and uncontrollable. Internal consistency (Cronbach’s *α*) in the present sample was 0.82.

##### Health related quality of life [53]

The KIDSCREEN-10 index allows a stable and reliable assessment of the health related quality of life. Internal consistency (Cronbach’s *α*) in the present sample was 0.86.

##### Childhood trauma questionnaire [CTQ; 54]

The CTQ is a validated screening instrument to retrospectively assess experiences of abuse (psychological, physical, sexual) and neglect (psychological, physical). Internal consistency (Cronbach’s *α*) in the present sample was 0.80.

### Statistical analyses

**Fig. 1:**
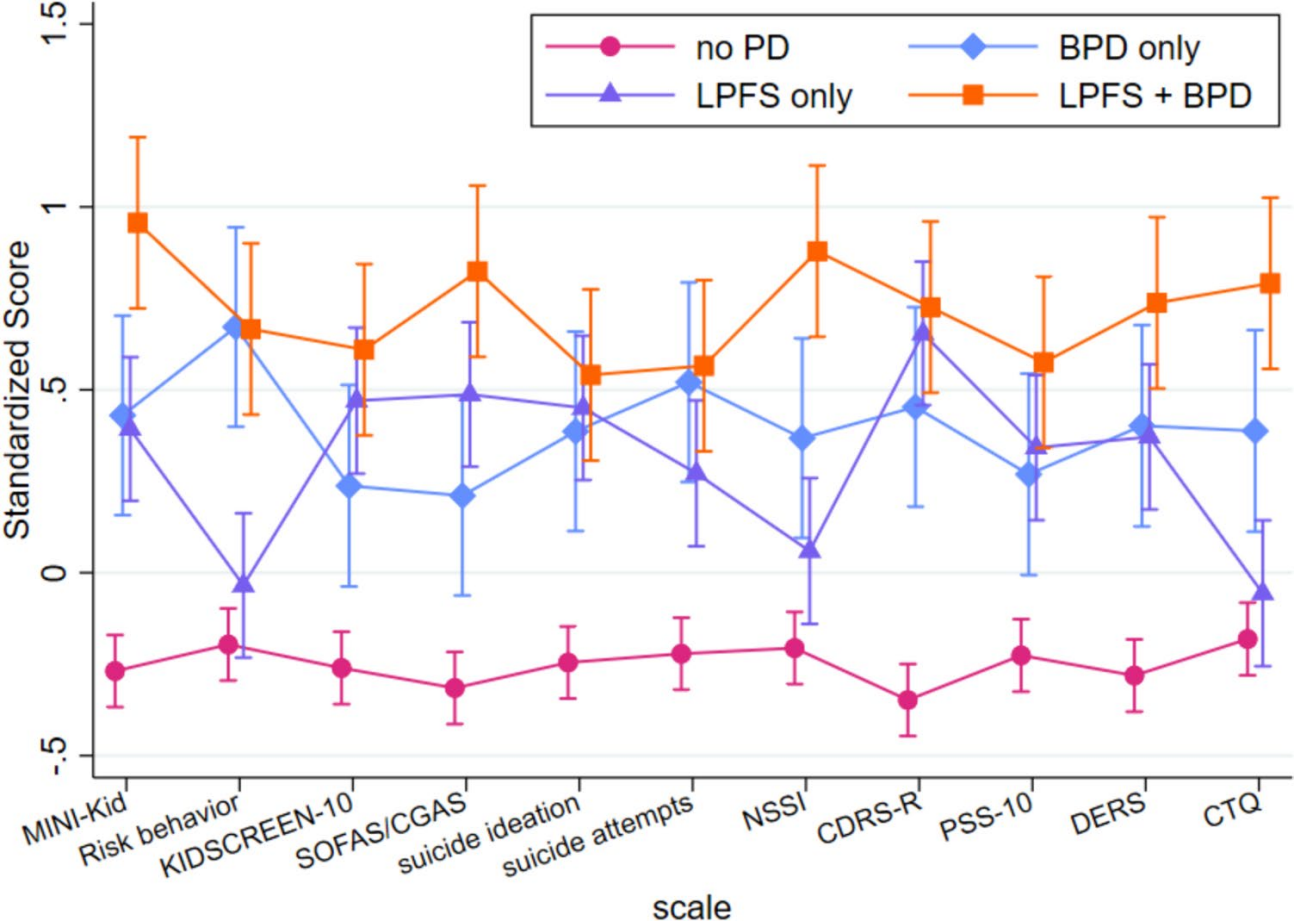
Profile of the four groups. *No PD* below threshold in both measures, *LPFS only* above threshold LPFS, *BPD only* above threshold BPD, *LPFS* + *BPD* above threshold in both measures. Abbreviations see Table 1

Descriptive information is presented in the form of means, standard deviations, frequencies, and percentages. Between-sample differences were tested using Wilcoxon rank-sum tests and *χ*^2^ tests. Pearson correlations between the number of facets of the LPFS with a value of ≥ 2 (diagnostic threshold), the number of fulfilled BPD criteria, and measures of psychopathology and psychosocial impairment were calculated. First, the concordance (aim 1) between LPFS and the BPD diagnosis was examined by categorizing individuals based on the diagnostic thresholds for PD according to the LPFS (i.e., ≥ 2 elements with mean value ≥ 2) and BPD (i.e., ≥ 5 out of 9 criteria), respectively. This resulted in four groups: No PD: below threshold in both measures, LPFS only: above threshold LPFS, BPD only: above threshold BPD, LPFS + BPD: above threshold in both measures. Next, differences in psychopathological/psychosocial profiles between the identified diagnostic groups were examined using a multilevel mixed-effects linear regression analysis (aim 2). The eleven variables (see Fig. 1) were combined into one model. Standardized scale scores served as the dependent variable. The right skewed scales (number of suicidal ideations, number of suicide attempts, frequency of NSSI) were transformed by square root transformation before standardizing. The KIDSCREEN and SOFAS/CGAS were reversed ensuring parity (i.e., low scores indicate a healthy subject). Group, Scale, Sample, Group × Scale interaction, and Sample × Scale interaction were included as fixed factors. The observations were grouped by subject, allowing for a random intercept. Post-hoc contrasts (i.e., comparisons between mean values) (a) between the no PD group and the three above diagnostic threshold groups (LPFS only, BPD only, LPFS + BPD), and (b) across the three above diagnostic threshold groups were undertaken, using the Wald test. Šidák-adjusted p values were computed to correct for 11 comparisons. All analyses are based on available observations, i.e. we included all 526 subject in the analyses and descriptives and used the available data to estimate the parameters and descriptives. Concretely, the missing data are ignored to calculate the descriptives. For the analyses we had 507 subject with BPD data and 502 subjects with BPD and LPFS data. In the mixed model the 502 subjects got 5481 observations (502 MINI-KID, 501 risk behavior, 495 KIDSCREEN-10, 500 SOFAS/CGAS, 501 suicide ideation, 499 suicide attempts, 498 NSSI, 502 CDRS-R, 495 PSS-10, 496 DERS, and 492 CTQ observations). Data quality was ensured following the quality measures described in the ethics. Plausibility and encoding checks were done when merging data from the two cohorts. The significance level was set to *α* = 0.05. All analysis were conducted using STATA SE 17.0 [55].

## Results

### Participants

Sample characteristics and descriptive results of all included variables are presented in Table 1. Comparison of the two samples revealed statistically significant differences for the following: Inpatients had a higher number of diagnoses and of above-threshold LPFS facets, as well as a higher LPFS total score, lower global level of functioning, more depressive symptoms, more difficulties in emotion regulation and more trauma experiences than outpatients. Outpatients had a higher frequency of NSSI, higher stress experience and lower health related quality of life, compared to inpatients.

#### Aim 1: Concordance of LPFS and BPD

A total of 64.1% (n = 322) of the sample was below the clinical threshold for either assessment (no PD), while 11.4% (n = 57) reached or exceeded the threshold for both assessments (LPFS + BPD). 16.1% (n = 81) fulfilled the LPFS threshold, but not the BPD threshold (LPFS only), and notably, 8.4% (n = 42) was above the threshold of BPD but not of LPFS (BPD only).

#### Aim 2: Psychopathological and psychosocial correlates

Correlation analyses showed small to large significant correlations between psychopathological and psychosocial variables and the number of facets of the LPFS with a value of ≥ 2 (*r* = 0.18–0.57, *p* < 0.001), as well as with the number of fulfilled BPD criteria (*r* = 0.24–0.52, *p* < 0.001) (see Table 1, supplementary materials). Here, we treat the STiP-5.1 as unidimensional. See supplementary material 2 for multifactor analyses.

A higher percentage of those with social anxiety disorder were found in the LPFS only group (46.9% vs. 19.1%, *χ*^2^ = 9.492, *p* = 0.002), while a higher percentage of conduct disorder (19.8% vs. 35.7%, *χ*^2^ = 3.588, *p* = 0.058) and PTSD (13.6% vs. 40.5%, *χ*^2^ = 11.125, *p* = 0.001) were found in the BPS only group.

The model fit of the mixed effects regression model was very good (χ^2^ (54) = 779.32, *p* < 0.001). Post-hoc comparisons revealed that patients who exceeded the diagnostic threshold on at least one measure (i.e., LPFS only, BPS only, LPFS + BPD) scored significantly higher on all psychopathological and psychosocial variables compared to the no PD group (see Fig. 1 for variable means by groups, and Table 2 for the statistical significance of differences in variable means between groups). The exceptions were traumatic experiences and risk behavior, which were of similar levels between the LPFS only group and the no PD group. Differences were most pronounced between the LPFS + BPD group and the no PD group.﻿

**Table 2:** Mean differences between the no PD group and the LPFS, the BPD and the LPFS + BPD group in measures of psychopathology or psychosocial impairment

	LPFS vs. no PD	BPD vs. no PD	LPFS + BPD vs. no PD
	Contrast (SE)	Contrast (SE)	Contrast (SE)
*Overall*	0.549 (0.064)***	0.640 (0.083)***	0.959 (0.073)***
*By scale*			
Diagnoses MINI-KID	0.620 (0.113)***	0.677 (0.148)***	1.188 (0.130)***
Risk behavior	0.205 (113) n.s	0.889 (0.148)***	0.900 (0.130)***
KIDSCREEN-10	0.805 (0.114)***	0.539 (0.149)**	0.932 (0.130)***
SOFAS/ CGAS	0.674 (0.113)***	0.463 (0.148)*	1.028 (0.130)***
Suicidal ideation week (SITBI)	0.710 (0.113)***	0.639 (0.148)***	0.799 (0.130)***
Suicide attempts year (SITBI)	0.495 (0.114)***	0.743 (0.148)***	0.789 (0.130)***
NSSI year (SITBI)	0.332 (0.114)*	0.609 (0.148)***	1.146 (0.130)***
CDRS-R	0.973 (0.113)***	0.786 (0.148)***	1.047 (0.130)***
PSS-10	0.650 (0.114)***	0.542 (0.149)**	0.874 (0.130)***
DERS-16	0.615 (0.114)***	0.661 (0.149)***	0.985 (0.130)***
CTQ	-0.035 (0.114) n.s	0.483 (0.149)*	0.837 (0.130)***

*p < 0.05, **p < 0.01, ***p < 0.001; negative values correspond to higher values in the second group; abbreviations see Table 1

In addition, post-hoc comparisons across the three above diagnostic threshold groups (i.e., LPFS only, BPD only, LPFS + BPD; see Table 3) revealed that the LPFS only group showed less risk behavior and reported fewer traumatic experiences compared to the BPD only group. Additionally, they had fewer diagnoses, less risk behavior and NSSI, and fewer traumatic experiences compared with the LPFS + BPD group. Finally, the BPD only group differed from the LPFS + BPD group in terms of fewer diagnoses and lower levels of NSSI, as well as higher level of psychosocial functioning.

**Table 3:** Mean differences across the groups LPFS, BPD, LPFS + BPD on measures of psychopathology or psychosocial impairment

	LPFS vs. BPD	LPFS vs. LPFS + BPD	BPD vs. LPFS + BPD
	Contrast (SE)	Contrast (SE)	Contrast (SE)
*Overall*	− 0.090 (0.101) n.s	0.408 (0.088)***	0.318 (0.103)**
*By scale*			
Diagnoses MINI-KID	− 0.057 (0.172) n.s	− 0.567 (0.156)**	− 0.510 (0.183)*
Risk behavior	− 0.684 (0.172)**	− 0.695 (0.156)***	− 0.011 (0.183) n.s
KIDSCREEN-10	0.266 (0.174) n.s	− 0.128 (0.157) n.s	− 0.394 (0.184) n.s
SOFAS/ CGAS	0.211 (0.172) n.s	− 0.354 (0.156) n.s	− 0.565 (0.183)*
Suicidal ideation week (SITBI)	0.070 (0.172) n.s	− 0.089 (0.156) n.s	− 0.160 (0.183) n.s
Suicide attempts year (SITBI)	− 0.248 (0.172) n.s	− 0.294 (0.157) n.s	− 0.046 (0.183) n.s
NSSI year (SITBI)	− 0.277 (0.172) n.s	− 0.814 (0.157)***	− 0.537 (0.183)*
CDRS-R	0.187 (0.172) n.s	− 0.075 (0.156) n.s	− 0.261 (0.183) n.s
PSS-10	0.108 (0.173) n.s	− 0.224 (0.157) n.s	− 0.332 (0.184) n.s
DERS-16	− 0.047 (0.173) n.s	− 0.371 (0.157) n.s	− 0.324 (0.184) n.s
CTQ	− 0.518 (0.174)*	− 0.872 (0.157)***	− 0.354 (0.184) n.s

*p < 0.05, **p < 0.01, ***p < 0.001; negative values correspond to higher values in the second group; abbreviations see Table 1

## Discussion

The goal of this study was twofold: First, to examine the concordance between the LPFS and a BPD diagnosis; and second, to explore differential psychopathological and psychosocial correlates in a representative clinical sample of adolescent in- and outpatients.

Overall, in the present sample, a proportion of 35.9% adolescent patients fulfill the diagnostic criteria of a PD, respectively of significant impairments in personality functioning, which is comparable to previous reports from clinical samples [1, 2]. The results of the present study regarding the concordance of the LPFS and BPD diagnosis were partly in line with our hypothesis: that is, 11.4% of patients exceeded the diagnostic threshold of both measures. This group aligns with the newly developed classification system of PD according to the AMPD and the ICD-11, which specifies the assessment of PD on the basis of a significant impairment in self- and interpersonal functioning (LPFS) in combination with pathological personality traits, with the option of a BPD pattern qualifier [24]. A total of 16.1% of patients reached diagnostic threshold for the LPFS, but not for BPD. This is consistent with the broader conceptualization of the LPFS as capturing the core of all PDs, not only of the subgroup of patients that meets the diagnostic criteria for BPD. The BPD qualifier thus serves to characterize a group of patients with symptoms beyond general PD. Another possible explanation is that the diagnostic thresholds of the two assessments for LPFS and BPD vary in sensitivity. However, and contrary to our hypothesis, a minority of patients (8.4%) exceeded only the BPD threshold but not the LPFS threshold. This diagnostic group does not correspond to the underlying theoretical model of the new conceptualization of PD in the AMPD and the ICD-11 that suggests an impairment in the level of personality functioning in all PDs [24, 25]. This result proposes a distinct diagnostic group characterized by BPD symptoms as assessed by the SCID-II, but low overall impairment in self- and interpersonal functioning (i.e., below the diagnostic threshold), as assessed by the LPFS in the STiP-5.1. Therefore, while they meet categorical BPD criteria, this group does not formally qualify as PD according to the new classification systems. The results might have been different if a measure assessing PD according to the ICD-11 operationalization had been used [e.g., PDS-ICD-11, 32], which also assesses self-harm as an aspect of PD severity. Consequently, more patients from the BPD only group with self-injurious/suicidal behavior would have been assigned to the LPFS + BPD group [32]. On the other hand, two further conclusions may be drawn from this result: First, the SCID-II BPD module may be able to more sensitively capture early emerging BPD symptoms in adolescents (i.e., predominately acute symptoms such as self-harm and impulsivity), and second, it may point to the necessity of a lower LPFS-threshold for adolescents. However, a different conclusion could be that this group may also be regarded as a distinct diagnostic group with severe emotional dysregulation and respective behaviors, but should not be labeled as PD in the future. Given that early intervention will still be important, caution is warranted to not oversee this important target group for diagnosis and subsequent treatment.

The correlation between the number of facets of the LPFS with a value of  ≥ 2 and the number of BPD criteria was moderate (*r* = 0.49). As expected, both constructs showed small to large significant correlations in the expected direction with measures of psychopathology and psychosocial impairments. Results confirm the high psychopathological comorbidity and psychosocial impairment of both BPD diagnosis [e.g., 3] and clinical impairment in personality functioning (LPFS) [e.g., 38]. The no PD group showed significantly lower levels of psychopathology and psychosocial impairment compared to the groups with clinically relevant PD pathology (LPFS only, LPFS + BPD, BPD only). Correlations between impairments in self- and interpersonal functioning with more fulfilled BPD criteria, as well as with more pronounced psychopathology, was also found by Weekers et al. in the only available other adolescent sample [41]. The highest overall comorbidity and impairment was found in individuals who exceeded the threshold of both measures, adding to the evidence that the most severely ill patients show clinically significant impairment in LPFS with the specifier BPD. The LPFS only group exhibits more internalizing disorders (such as social anxiety disorder), whereas the BPD only group appears to be characterized by externalizing disorders (such as conduct disorder). Moreover, the two groups were distinguishable in terms of higher risk behavior and more traumatic experiences in the BPD only group. It has previously been shown that high risk behavior and conduct disorders are common in full-syndrome and subthreshold BPD [e.g., 1, 5], and that traumatic experiences are closely associated with BPD [e.g., 10]. Our results might refer to a subgroup of adolescents who may be best described by the newly established diagnosis complex post-traumatic stress disorder (CPTSD), which is characterized by BPD features such as emotion dysregulation, disturbances in identity and relationships in addition to the core PTSD symptoms [56].

To our knowledge, this is only the second study that systematically assessed impairments in personality functioning (LPFS) according to the AMPD in adolescents. Data were assessed by structured clinical interviews which entails several advantages over self-report [57]. For the first time, this study directly compared the alternative approach to conceptualize personality pathology with BPD diagnosis. The present sample includes a large group of in- and outpatients recruited in a naturalistic clinical setting and thus, can be considered as representative. Nevertheless, several limitations should be considered: First, no healthy control group of adolescents has been recruited. Second, criterion B (pathological personality traits) of the AMPD was not assessed, which would be necessary for a hybrid diagnosis of PD according to the AMPD. Third, the LPFS was assessed based on a validated semi-structured clinical interview only, while a multimethod assessment combining self-report with structured interview is recommended [57]. Fourth, interrater reliability has not been tested in this sample, however has been confirmed before [58]. Sixth, even if the LPFS offers a more developmentally sensitive approach to PD assessment, the STiP-5.1 interview was developed for adults and (although it was validated in this age group) not specifically adapted for adolescents.

The results of the present study have several implications for clinical practice and future research. First, validity of criterion A (LPFS) in the new conceptualization of PD in the AMPD was supported and its potential for the application in adolescent samples was underlined. This is in line with the demand for a dimensional and more developmentally sensitive conceptualization of PD. The dimensional assessment of impairment in personality functioning may be a more suitable approach to identify early symptoms of general psychopathology, and high risk of developing a PD in adolescents than the current categorical classification. With its dimensional structure, the AMPD may crucially contribute to the implementation of diagnosis and treatment of PDs in adolescence, since it allows for a developmental perspective, and may thus reduce skepticism in clinicians [21]. In addition to providing diagnostic information about the presence and severity of a PD, the STiP-5.1 provides information about the areas of self and interpersonal functioning in which a person is experiencing difficulties. This information may be useful for treatment planning, as it allows the content to be individually tailored (e.g. in the context of a modular approach). The present results support the usefulness of assessing both LPFS and BPD pattern qualifier as suggested by the ICD-11 [24]. Subsequent studies should address the question of the appropriate threshold of the LPFS in adolescents and its cross-cultural validity. It may be useful to consider subthreshold impairment of personality functioning as in BPD, where subthreshold forms are similarly associated with psychopathology and impairment as full-syndrome forms [e.g., 22]. Furthermore, stability and predictive value of impairment in personality functioning in adolescence should be the target of longitudinal investigations. Finally, future research and clinical practice will need to find a way to adequately consider the phenotype of adolescent BPD without significant impairment in personality functioning, which is not captured by the new classification systems.

## Conclusion

Both a significant impairment in personality functioning (LPFS), as well as a BPD diagnosis were similarly associated with high levels of psychopathology and psychosocial impairment. Most severely ill patients showed clinically significant impairment in LPFS with the specifier BPD according to the ICD-11. Unexpectedly, a group of adolescents was identified with low impairment in personality functioning (LPFS), but full-syndrome BPD, characterized by higher risk behavior and more traumatic experiences compared to the LPFS only group.

### Key points and relevance

- *What is known:* BPD in adolescence is associated with high comorbidity and psychosocial impairment. Little is known about the relevance and validity of the alternative conceptualization of PD according to the LPFS in adolescents.
- *What’s new:* Significant impairment in the LPFS is similarly associated with psychopathology and psychosocial impairment as BPD diagnosis. A BPD only group was identified, which differs from the LPFS group in respect of higher risk behavior and more traumatic experiences, and does not formally qualify as PD in the new AMPD classifications.
- *What’s relevant for clinical practice:* The LPFS provides meaningful diagnostic information in clinical adolescent samples. Most severely ill adolescents were characterized by significant impairments in personality functioning (LPFS) with specific BPD diagnosis. However, there is a need to clarify how to deal with the BPD only individuals who do not fulfill formal PD diagnosis according to AMPD.

## Supplementary Information

Below is the link to the electronic supplementary material.

Supplementary file1 (DOCX 16 KB)

Supplementary file2 (DOCX 16 KB)

## Data Availability

Data is available from the corresponding author upon request.
