# Supplementary material for: Comparing the new concept of impairment in personality functioning with borderline personality disorder: differential psychosocial and psychopathological correlates in a clinical adolescent sample
Source: Eur Child Adolesc Psychiatry. 2024 Aug 16;34(3):1183–93. doi: 10.1007/s00787-024-02555-y (PMC11909016; doi:10.1007/s00787-024-02555-y)
Supplement: Supplementary file 1 — Supplementary file1 (DOCX 16 KB) [file 787_2024_2555_MOESM1_ESM.docx]

**Supplementary material 1**

**Table 1.** Correlations between LPFS, BPD and psychopathology/ psychosocial impairment.

|  | LPFS^1^  *r*, *p*, [CI] | BPD^2^  *r*, *p*, [CI] |
| --- | --- | --- |
| LPFS | 1.00 | .49  <.001, [0.41,0.56] |
| BPD | .49  <.001, [0.41,0.56] | 1.00 |
| Age (years) | .00  .965, [-0.09,0.10] | .24  <.001, [0.16,0.32] |
| Diagnoses MINI-KID | .42  <.001, [0.34,0.50] | .52  <.001, [0.45,0.58] |
| Risk behavior | .19  <.001, [0.10,0.28] | .40  <.001, [0.32,0.47] |
| KIDSCREEN-10 | -.32  <.001, [-0.40,-0.23] | -.37  <.001, [-0.44,-0.29] |
| SOFAS/ CGAS | -.47  <.001, [-0.54,-0.40] | -.38  <.001, [-0.45,-0.30] |
| Suicidal ideation (SITBI) | .31  <.001, [0.22,0.39] | .32  <.001, [0.24,0.39] |
| Suicide attempts (SITBI) | .25  <.001, [0.16,0.34] | .34  <.001, [0.26,0.41] |
| Number NSSI (SITBI) | .22  <.001, [0.12,0.31] | .41  <.001, [0.33,0.48] |
| CDRS-R | .47  <.001, [0.39,0.54] | .46  <.001, [0.39,0.53] |
| PSS-10 | .26  <.001, [0.16,0.34] | .36  <.001, [0.28,0.43] |
| DERS-16 | .41  <.001, [0.32,0.48] | .44  <.001, [0.37,0.51] |
| CTQ | .21  <.001, [0.12,0.30] | .38  <.001, [0.30,0.45] |

*Notes.* The right skewed scales (number of suicidal ideations, number of suicide attempts, number of NSSI) were transformed by taking the square root before standardizing. BPD = Borderline Personality Disorder; LPFS = Level of Personality Functioning Scale; MINI-KID = Mini-International Neuropsychiatric Interview for Children and Adolescents; SOFAS = Social and Occupational Functioning Assessment Scale; CGAS = Children's Global Assessment Scale; KIDSCREEN-10 = Health Related Quality of Life; SITBI-G = Self-Injurious Thoughts and Behavior Interview – German Version; CDRS-R = Children’s Depression Rating Scale – Revised; PSS-10 = Perceived Stress Scale; DERS-16 = Difficulties in Emotion Regulation Scale, 16-item version; CTQ = Childhood Trauma Questionnaire.
