# Supplementary material for: Comparing the new concept of impairment in personality functioning with borderline personality disorder: differential psychosocial and psychopathological correlates in a clinical adolescent sample
Source: Eur Child Adolesc Psychiatry. 2024 Aug 16;34(3):1183–93. doi: 10.1007/s00787-024-02555-y (PMC11909016; doi:10.1007/s00787-024-02555-y)
Supplement: Supplementary file 2 — Supplementary file2 (DOCX 16 KB) [file 787_2024_2555_MOESM2_ESM.docx]

**Supplementary material 2**

We treat the structure of STiP-5.1 as unidimensional, i.e. we sum the dichotomized facets. Three exploratory factor analyses driven by the four elements of the STiP-5.1 (4-factor model: four factors measured each by the corresponding three items of each element; 2-factor model: two factors measured by the corresponding six items of the domains; 1-factor analysis: one factor measured by all 12 facets) show high correlations between the factors in the multifactor models and thus legitimate our approach (Table 1). Although, the four factor model shows the best fit indices (Table 2).

**Table 1.** Correlations between factors

| **Model** | **Between** | **Correlation** | **95%CI** | **p-value** |
| --- | --- | --- | --- | --- |
| 2-factor | F1 <-> F2 | 0.74 | [0.68,0.80] | <0.001 |
| 4-factor | F1 <-> F2 | 0.84 | [0.78,0.91] | <0.001 |
| 4-factor | F1 <-> F3 | 0.59 | [0.50,0.69] | <0.001 |
| 4-factor | F1 <-> F4 | 0.67 | [0.60,0.75] | <0.001 |
| 4-factor | F2 <-> F3 | 0.72 | [0.63,0.81] | <0.001 |
| 4-factor | F2 <-> F4 | 0.68 | [0.60,0.76] | <0.001 |
| 4-factor | F3 <-> F4 | 0.79 | [0.71,0.86] | <0.001 |

*Notes.* For the 2-factor model, F1 is measured by the 6 facets of the domain self-functioning; F2 is measured by the 6 facets of the domain interpersonal functioning. For the 4-factor model, F1 is measured by the 3 facets of the element identity; F2 is measured by the 3 facets of the element self-direction; F3 is measured by the 3 facets of the element empathy; F4 is measured by the 3 facets of the element intimacy.

**Table 2.** Fit indices of the three factor analysis

|  | **4-factor model** | **2-factor model** | **1-factor model** |
| --- | --- | --- | --- |
| Number of factors | 4 | 2 | 1 |
| degree of freedom | 48 | 53 | 54 |
| chi^2^ | 142.4 | 220.4 | 392.2 |
| RMSEA | 0.063 | 0.079 | 0.112 |
| CFI | 0.955 | 0.920 | 0.838 |
| TLI | 0.938 | 0.900 | 0.802 |
| SRMR | 0.040 | 0.046 | 0.066 |
